# Investigating the factors affecting myopia in retinopathy of prematurity after laser treatment

**DOI:** 10.1186/s40942-023-00456-x

**Published:** 2023-04-12

**Authors:** Shotaro Asano, Tatsuya Inoue, Kana Kure, Marie Kitano, Asahi Fujita, Miyuki Nagahara, Ryo Asaoka, Ryo Obata

**Affiliations:** 1 Department of Ophthalmology, The University of Tokyo, Tokyo, Japan; 2 Department of Ophthalmology, Asahi General Hospital, Asahi, Chiba Japan; 3 Department of Ophthalmology and Micro-Technology, Yokohama City University, Kanagawa, Japan; 4 Department of Ophthalmology, Seirei Hamamatsu General Hospital, Shizuoka, Hamamatsu Japan; 5 Seirei Christopher University, Shizuoka, Hamamatsu Japan

**Keywords:** Retinopathy of prematurity, Laser treatment, Myopia

## Abstract

**Background:**

We investigated the effect of the number of laser shots applied on the myopic variables to elucidate the mechanism of myopia development in laser-treated retinopathy of prematurity (ROP) eyes.

**Methods:**

A total of 33 eyes of 17 infants with ROP who underwent laser treatment were included in the analysis. Cycloplegic retinoscopic refraction testing was carried out and the spherical equivalent (SE) was calculated. Relationships between SE and various variables (including the number of laser shots applied) were examined. In addition, an age-matched control group without ROP was prepared and ocular structural parameters were compared.

**Results:**

Although there was no statistical difference in axial length (AL) between two groups (p = 0.88), SE was significantly more myopic in the ROP group (p < 0.001). SE was associated with AL, corneal refraction (CR), and crystalline lens power (CLP) in the ROP group. Of these three factors (AL, CR, and CLP), CLP and the number of laser shots applied were significantly correlated (p = 0.003); however, no correlations were observed between the number of laser shots and AL or CR (p = 0.15 and 0.10, respectively). Very similar tendency was observed in the analysis of the difference between right and left eyes in each child.

**Conclusions:**

In laser-treated ROP eyes, AL, CR, and CLP were related to the degree of myopia. Moreover, the number of shots applied also affected the myopic status in laser-treated ROP eyes. Among AL, CR, and CLP, only CLP was correlated with the laser shots applied.

## Background

Retinopathy of prematurity (ROP) is a retinal ischemic disease caused by the development of abnormal retinal vessels in prematurely born infants [1]. Previous studies revealed that laser treatment had no impact on axial length (AL) [2–6], although spherical equivalent (SE) of laser-treated eyes with ROP significantly shifted towards myopia [3–7]. An accepted hypothesis is that laser treatment leads to a destruction of the peripheral retina and parts of the choroid, which may interfere with a possible disruption in the development of the anterior segment and may potentially lead to higher rate of myopia [1, 8]. Importantly, Connolly et al. investigated the myopic status in ROP eyes treated with laser and cryotherapy in a randomized controlled clinical trial. They suggested that the excess crystalline lens power (CLP) caused by the tissue destruction associated with laser treatment was another dominant component of the myopia in those eyes [9]. In particular, in eyes with laser treatment, CLP bore a strong correlation to refractive outcomes, with a correlation coefficient of 0.885 [9].

We recently reported that there was a correlation between laser shots applied and the degree of myopia in eyes with laser-treated ROP at the age of 3 years [10]. This finding was consistent with a previous study showing eyes treated with laser treatment had a higher prevalence of myopia than eyes treated with anti-vascular endothelial growth factor (VEGF) monotherapy [11]. However, our previous study was conducted without including other detailed parameters related to myopia such as AL, CLP, corneal refraction (CR), and anterior chamber depth (ACD).

In the current study, we investigated the effect that the number of applied laser shots had on these detailed myopic variables in eyes with ROP after laser treatment. In addition, an age-matched control group without ROP was prepared and various ocular structural parameters were compared between the eyes with laser-treated ROP and the control eyes.

## Methods

This study, conducted with the approval of the Institutional Review Board of the University of Tokyo (approval number: 2217), adhered to the tenets of the Declaration of Helsinki. Written informed consent was obtained from the parents of each of the participants, and we retrospectively reviewed the medical records at the outpatient clinic of Tokyo University Hospital.

### Study population

In the ROP group, we included eyes with ROP that had histories of laser treatment and that underwent cycloplegic retinoscopic refraction testing between January 1, 2004 and December 31, 2018 (between 3 and 10 years of age). Eyes with apparent eye diseases other than ROP and children who had undergone any intraocular surgery or received any anti-vascular endothelial growth factor (VEGF) therapy were excluded from the ROP group.

For comparison, the following were assigned as the age-matched control group: (i) infants with no known eye disease who visited the University of Tokyo Hospital within the same period for regular check-up; (ii) infants who underwent cycloplegic retinoscopic refraction testing; and (iii) infants between 3 and 8 years of age. As a result, 33 eyes of 17 infants and 14 eyes of 7 infants were enrolled as the ROP group and the control group, respectively.

### Treatment and clinical examination

All of the eyes with ROP had undergone diode laser treatment under sedation. The laser-treated eyes were in either the threshold or prethreshold stage of ROP according to the ETROP guideline [12]. The laser treatments were performed by one of two ophthalmologists (T.I. and M.N.) in all eyes to the peripheral avascular retina to alleviate the dilation and tortuosity of retinal vessels and vasoproliferation. The ophthalmological assessments including slit lamp examinations, intraocular pressure measurement, and fundoscopy were performed as routine check-up. Cycloplegic retinoscopic refraction testing was carried out after cycloplegia with atropine or cyclopentolate hydrochloride eye drops, and the spherical equivalent (SE) was calculated as the spherical power + ½ of the cylindrical power. At the same time, corneal refraction (CR), AL, and anterior chamber depth (ACD) were measured using optical biometry (OA-2000; Tomey, Nagoya, Japan). CLP was calculated using the modified Sanders-Reetzlaff-Kraff formula according to previous studies [9, 13]. In the ROP group, birth weight was also collected.

### Statistical analysis

Mann-Whitney U test was used to compare demographics of each group, with the exception of sex ratio and laterality, which was evaluated using the Chi-squared test.

In the ROP group, firstly, the relationship between SE and the number of laser shots were analyzed with the Person’s correlation test. Then, the relationship between SE and the variables of age, sex, birth weight, CR, AL, ACD, and CLP were investigated using the linear mixed model. The linear mixed model is equivalent to ordinary linear regression in that the model describes the relationship between the predictor variables and a single outcome variable. However, standard linear regression analysis makes the assumption that all observations are independent of each other. In the current study, measurements were nested within subjects and thus were dependent of each other. Ignoring this grouping of the measurements will result in the underestimation of standard errors of regression coefficients. The linear mixed model adjusts for the hierarchical structure of the data, modeling in a way in which measurements are grouped within subjects to reduce the possible bias of including both eyes of one patient [14, 15]. The model selection was then performed to identify the optimal linear mixed model for SE using the second-order bias-corrected Akaike information criterion (AICc) index, from all 2^7^ patterns consisted of 7 variables (age, sex, birth weight, CR, AL, ACD, and CLP). The AIC is an established statistical measurement used in model selection, and the AICc is a corrected version of the Akaike information criterion index, which provides an accurate estimation even when the sample size is small [16]. The selected variables through the model selection were regarded as statistically significant [17]. In addition, correlations between the number of laser shots applied and CR, AL, ACD, and CLP were investigated using the Pearson’s correlation test.

Thereafter, the anatomical difference between right and left eyes were calculated using both eyes with ROP from same infants (16 pairs) to exclude background characteristics. First, the difference in the SE (ΔSE, calculated as the value in the right eye minus that in the left eye) was calculated. Then the association between ΔSE and the variables of age, sex, birth weight, and the differences in CR (ΔCR), AL (ΔAL), ACD (ΔACD), and CLP (ΔCLP) between the two eyes were investigated using linear regression analysis, and the model selection using the AICc index, respectively; the optimal model was identified from 2^7^ patterns consisted of 7 variables (age, sex, birth weight, ΔCR, ΔAL, ΔACD, and ΔCLP). Moreover, correlations between the differences in number of laser shots applied between the two eyes (ΔShot) and ΔCR, ΔAL, ΔACD, and ΔCLP were calculated using Pearson’s correlation test.

All statistical analyses were carried out using *R* statistical software (version 3.6.3; http://www.r-project.org/). P values in multiple comparisons were corrected using the Hochberg correction.

## Results

The demographic data of enrolled eyes are shown in Table 1. The ROP group consisted of 6 eyes (18.2%) in Stage 2, and 27 eyes (81.8%) in Stage 3, which presented in Zone I in 4 eyes (12.1%) or Zone II in 29 eyes (87.9%) (Table 2). The mean birth weight in the ROP group was 771.9 ± 253.2 g (mean ± standard deviation). Cycloplegic retinoscopic refraction testing was carried out at 5.0 ± 1.5 years of age in the ROP group, and at 5.20 ± 1.9 years in the control group, respectively (p = 0.45). Although there was no statistical difference in AL between the ROP group and the control group (p = 0.88; Mann-Whitney U test), the SE value was significantly more negative (more myopic) in the ROP group compared to the control group (p < 0.001; Mann-Whitney U test). Significantly greater CR, shallower ACD, and greater CLP were observed in the ROP group compared to the control group (p < 0.001, p < 0.001, and p = 0.004, respectively; Mann-Whitney U test).

**Table 1:** Demographics of the subjects

Parameter		ROP^a^	Control	P value
Laterality (right/left)		17/16	7/7	1
Age, years		5.03 ± 1.5	5.20 ± 1.9	0.45
Sex (male/female)		9/8	2/5	0.52
LogMAR		0.20 ± 0.25	0.04 ± 0.08	0.02
SE^b^, D		-3.02 ± 4.96	1.41 ± 2.37	< 0.001
	Sphere, D	-2.48 ± 4.78	1.61 ± 2.35	0.007
	Cylinder, D	-1.07 ± 1.32	-0.39 ± 1.31	0.003
CR^c^, D		47.1 ± 1.8	44.1 ± 1.6	< 0.001
	K1, D	46.0 ± 1.9	43.2 ± 1.3	< 0.001
	K2, D	48.1 ± 1.9	44.9 ± 1.9	< 0.001
ACD^d^, mm		3.11 ± 0.36	3.45 ± 0.21	< 0.001
AL^e^, mm		22.1 ± 1.5	22.0 ± 1.0	0.88
CLP^f^, D		23.8 ± 5.2	20.0 ± 1.8	0.004

^a^: ROP, retinopathy of prematurity; ^b^: SE, spherical equivalent; ^c^: CR, corneal refraction; ^d^: ACD, anterior chamber depth; ^e^: AL, axial length; ^f^: CLP, crystalline lens power.

**Table 2:** Zones and Stages in the ROP group

	Number of eyes	Number of laser shots applied	Spherical Equivalent
Stage 2	6 (18.2%)	1533.7 ± 651.6	-6.67 ± 7.3
Stage 3	27 (81.8%)	870.5 ± 439.2	-2.21 ± 4.0
Zone 1	4 (12.1%)	1439.5 ± 641.6	-7.91 ± 8.7
Zone 2	29 (87.9%)	929.2 ± 505.2	-2.3 ± 4.0

**Fig. 1:**
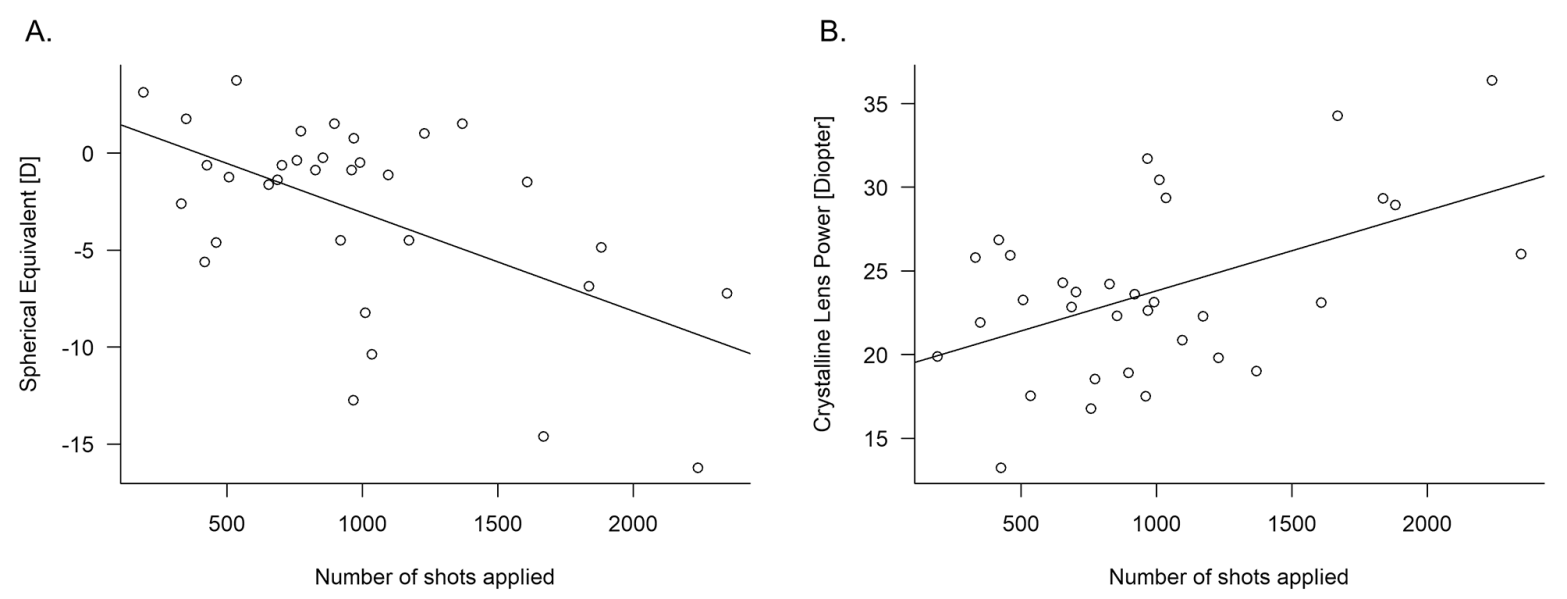
(**A**) Relationship between the number of laser shots applied and SE. A　significant relationship was observed between the number of laser shots applied and SE (coefficient = -0.55, p < 0.001; Person’s correlation test). (**B**) Correlation between the number of laser shots applied and CLP. There was a significant correlation between the number of laser shots applied and CLP (p = 0.003, r = 0.50; Pearson’s correlation test) SE: spherical equivalent, CLP: crystalline lens power

In the ROP group, SE showed significant negative correlation with the number of laser shots applied (coefficient = -0.55, p < 0.001; Person’s correlation test, Fig. 1A). As shown in Table 3, the optimal linear model identified for SE in ROP eyes was as follows; SE = 77.2–1.78 (Standard Error [Stderr] = 0.021, p < 0.001) x AL – 0.52 (Stderr = 0.021, p < 0.001) x CR – 0.68 (Stderr = 0.007, p < 0.001) x CLP (AICc = 3.7).

**Table 3:** Relationships between SE^a^ and each of age, sex, birth weight, CR^b^, AL^c^, ACD^d^, and CLP^e^ in laser-treated eyes with ROP^f^

Parameter	Univariate analysis			Optimal model		
	Coefficient	Stderr^g^	P value	Coefficient	Stderr^g^	P value
Age	0.044	0.056	0.44	N.S.^h^		
Sex	0.24	2.33	0.92	N.S.^h^		
Birth weight	0.0087	0.0042	0.054	N.S.^h^		
CR^b^	-0.76	0.550	0.19	-0.52	0.021	< 0.001
AL^c^	-2.18	0.35	< 0.001	-1.78	0.021	< 0.001
ACD^d^	8.52	2.72	0.0069	N.S.^h^		
CLP^e^	-0.85	0.093	< 0.001	-0.68	0.007	< 0.001

^a^: SE, spherical equivalent; ^b^: CR, corneal refraction; ^c^: AL, axial length; ^d^: ACD, anterior chamber depth; ^e^: CLP, crystalline lens power; ^f^: ROP, retinopathy of prematurity; ^g^: Stderr, standard error; ^h^: N.S., not selected.

Pearson’s correlation test results showed significant correlation between the number of laser shots applied and CLP (coefficient = 0.50, p = 0.003, Fig. 1B). In contrast, no correlations were observed between the number of laser shots applied and each of AL, ACD, and CR (p = 0.15, 0.65, and 0.10, respectively; Pearson’s correlation test).

As shown in Table 4, the optimal linear model for ΔSE was; ΔSE = 0.006–1.74 (Stderr = 0.029, p < 0.001) x ΔAL – 0.58 (Stderr = 0.049, p < 0.001) x ΔCR – 0.69 (Stderr = 0.014, p < 0.001) x ΔCLP (AICc = -6.9).

**Table 4:** Relationships between ΔSE and each of age, sex, birth weight, ΔCR, ΔAL, ΔACD, and ΔCLP in laser-treated eyes with ROP

Parameter	Univariate analysis	Optimal model				
	Coefficient	Stderr^g^	P value	Coefficient	Stderr^g^	P value
Age	-0.063	0.056	0.28	N.S.^h^		
Sex	-0.36	1.76	0.84	N.S.^h^		
Birth weight	0.0028	0.0034	0.44	N.S.^h^		
ΔCR^b^	-2.02	1.04	0.072	-0.58	0.049	< 0.001
ΔAL^c^	-2.17	0.39	< 0.001	-1.74	0.029	< 0.001
ΔACD^d^	21.2	8.51	0.026	N.S.^h^		
ΔCLP^e^	-0.91	0.24	0.0020	-0.69	0.014	< 0.001

^a^: ΔSE, differences in spherical equivalent; ^b^: ΔCR, differences in corneal refraction; ^c^: ΔAL, differences in axial length; ^d^: ΔACD, differences in anterior chamber depth; ^e^: ΔCLP, differences in crystalline lens power; ^f^: ROP, retinopathy of prematurity; ^g^: Stderr, standard error; ^h^: N.S., not selected.

The Pearson’s correlation test identified a significant correlation between the difference in the number of laser shots applied and ΔCLP (coefficient = 0.59, p = 0.017). There were no correlations between the difference in the number of laser shots applied and each of ΔAL, ΔACD, and ΔCR (p = 0.21, 0.20, and 0.86, respectively; Pearson’s correlation test).

## Discussion

In the current study, various parameters related to myopia were compared between laser-treated ROP (33 eyes of 17 infants) and age-matched control groups (14 eyes of 7 infants). As a result, there were significant differences in SE, CR, ACD, and CLP between the ROP group and the control group, despite the non-significantly different AL.

In laser-treated ROP eyes, the amount of laser treatment was significantly associated with the degree of myopia (Fig. 1A), and CR, AL, and CLP were significantly related to SE (Table 3). Furthermore, ΔCR, ΔAL, and ΔCLP were associated with ΔSE (Table 4). Finally, the number of laser shots applied was associated with CLP, but not with AL and CR, and the difference in the number of laser shots was associated with only ΔCLP.

Previous studies have compared myopic changes in eyes with ROP. Algawi et al. suggested that the number of eyes encountered myopia was significantly smaller in the laser group (40%) than the cryotherapy group (92%) [18]. Knight-Nanan et al. reported that 94.1% of a cryotherapy-treated group were myopic while 45.5% were myopic in a laser-treated group after 3 years of follow-up [19]. Connolly et al. reported that those treated with laser had a mean SE of -4.48 D, whereas this value was − 7.65 D in the cryotherapy-treated eyes [9]. Consistent with previous reports, our current results suggested laser-treated ROP eyes were significantly more myopic than the age-matched eyes. Moreover, in the current study, the degree of myopia in ROP eyes was negatively correlated with the number of laser shots applied (Fig. 1A), and significantly associated with CR, AL and CLP (Tables 3 and 4). Interestingly, AL was selected as an explanatory variable for the degree of myopia in laser-treated ROP, whereas there was no significant difference in AL between the ROP group and the control. Our result suggested AL is a non-negligible component of myopia-related parameters in laser-treated ROP eyes similar to normal eyes, however further study is needed to investigate the temporal change of AL in ROP children.

Connolly et al. also indicated CLP was more increased in cryotherapy-treated eyes than in laser-treated eyes, which was attributed to the greater tissue destruction of peripheral retina caused by cryotherapy, which disrupted the maturation of the anterior segment; i.e. zonules, the ciliary body, and the lens [9]. However, no reports investigated which refractive parameters were associated with the number of laser shots and influenced the degree of myopia. In the current study, we identified that CLP (but not CR and AL) was affected by the number of laser shots applied (p = 0.003, Fig. 1B**)**. There was also a significant association between the difference in the number of laser shots applied and ΔCLP. Taken together, it was suggested that the degree of myopia in laser-treated ROP eye is increased predominantly through increased CLP.

Lee et al. reported that ACD was significantly shallower in laser-treated ROP eyes than in eyes treated using anti-VEGF [8]. In the current study, ACD was significantly shallower in laser-treated ROP eyes compared to control eyes (Table 1). In contrast, there was no significant relationship between ACD and the degree of myopia as a result of multivariate analysis and model selection (Table 2). These contradicting results may be due to the different calculation of CLP in the current study; following previous studies [9, 13], the CLP was calculated using the modified Sanders-Reetzlaff-Kraff formula in which only the values of AL, keratometry measurement, and the refractive error were used. In other words, CLP was calculated including the effect of ACD, and indeed CLP was significantly correlated with ACD. In future, CLP estimation (excluding the effect of ACD) would be needed for laser-treated ROP eyes.

Recently, laser treatment and anti-VEGF therapy have been widely applied for ROP in many countries [11, 12, 20–22]. Indeed, anti-VEGF therapy has been shown to achieve better refractive and anatomic outcomes in general than laser treatment [8]. As shown in the current study, and consistent with a previous report [11], anti-VEGF therapy may be better than laser treatment in reducing subsequent progression of myopia, because it is unlikely that anti-VEGF therapy causes tissue destruction compared with laser treatment. However, careful consideration is still needed when choosing between the two treatments as anti-VEGF therapy is reportedly associated with delayed vascularization compared to laser treatment [8]. Mintz-Hittner reported that the mean time to recurrence was 16.0 weeks after anti-VEGF therapy, as compared with 6.2 weeks after laser treatment [23]. In other words, treatment cannot be regarded as successfully completed until no active disease or clinically significant tractional elements are confirmed on completion of vascularization [23].

There are several limitations in this study. Firstly, as the laser treatment was conducted by two clinicians, performance of the laser procedure (such as laser power and duration) was not completely consistent when applied among the patients. This discrepancy may be attributed to a non-negligible effect on the degree of tissue destruction caused by the laser treatment, resulting in the difference in development of myopia. In addition, the refractive measurements were performed in patients of different ages. The myopic change might partly progress with the natural course of axial length elongation, thus further studies should be performed to investigate myopia-related parameters at the same age in ROP and control eyes. A prospective study with rigid protocols will help us to clarify the current study’s results. Besides, due to the limited number of eyes included in the study, we could not conduct subgroup analysis with zones and stages of ROP. It is reasonable to assume that the difference in the severity of disease and the location of retina affected by the disease may cause changes in the amount of VEGF production, which might lead to ocular structural changes. However, in the current study, there were no statistical differences in SE between Zone 1 and 2, and Stage 2 and 3 (p = 0.36 and 0.22, Wilcoxon signed rank test). We suspect that those results might be due to the limited number of eyes included in the current study. Similarly, use of anti-VEGF therapy may also affect the anatomical structures of ROP eyes. This study did not include ROP patients treated with anti-VEGF agents, therefore it is important to compare the refractive errors between eyes treated with anti-VEGF agent and laser photocoagulation in order to clarify the effect of laser ablation on the myopic shift. A future study with larger sample size may help to reveal those issues in details.

In conclusion, the SE value was significantly more myopic in the laser-treated ROP group compared to the control group. In the laser-treated ROP eyes, the SE value was related to CR, AL, and CLP. The number of laser shots was associated with CLP, but not with AL and CR, suggesting CLP was a primary component of the refractive error of laser-treated ROP eyes.

## Data Availability

The dataset used and analyzed during the current study is available from the corresponding author on reasonable request.

## List of abbreviations

ROP: Retinopathy of prematurity
AL: Axial length
CLP: Crystalline lens power
VEGF: Vascular endothelial growth factor
CR: Corneal refraction
ACD: Anterior chamber depth
SE: Spherical equivalent
AIC: Akaike information criteria
